# Supplementary material for: Venomix: a simple bioinformatic pipeline for identifying and characterizing toxin gene candidates from transcriptomic data
Source: PeerJ. 2018 Jul 31;6:e5361. doi: 10.7717/peerj.5361 (PMC6074769; doi:10.7717/peerj.5361)
Supplement: Supplemental Information 6 [file peerj-06-5361-s006.gz › FinalOutput_1E-20/Techylectin-like_protein_1/finaltree.pdf]

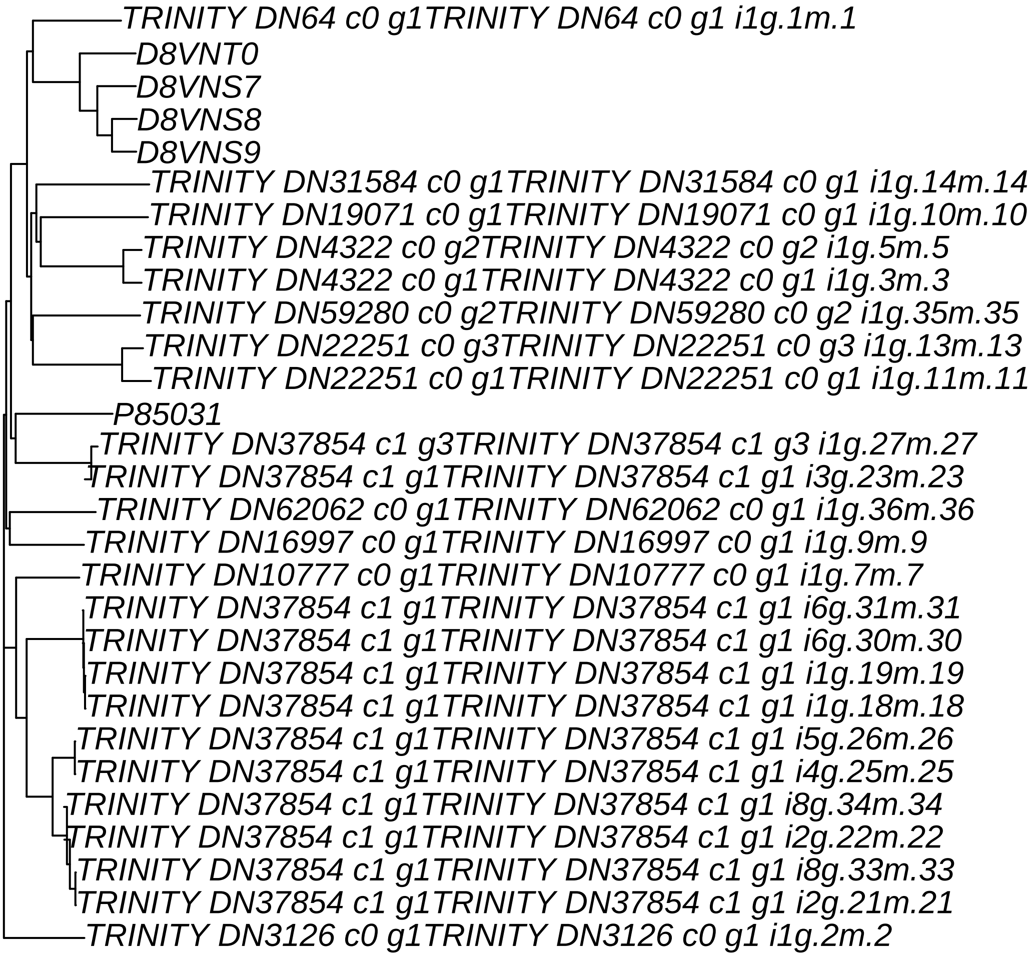
TRINITY DN64 c0 g1TRINITY DN64 c0 g1 i1g.1m.1
D8VNT0
D8VNS7
D8VNS8
D8VNS9
TRINITY DN31584 c0 g1TRINITY DN31584 c0 g1 i1g.14m.14
TRINITY DN19071 c0 g1TRINITY DN19071 c0 g1 i1g.10m.10
TRINITY DN4322 c0 g2TRINITY DN4322 c0 g2 i1g.5m.5
TRINITY DN4322 c0 g1TRINITY DN4322 c0 g1 i1g.3m.3
TRINITY DN59280 c0 g2TRINITY DN59280 c0 g2 i1g.35m.35
TRINITY DN22251 c0 g3TRINITY DN22251 c0 g3 i1g.13m.13
TRINITY DN22251 c0 g1TRINITY DN22251 c0 g1 i1g.11m.11
P85031
TRINITY DN37854 c1 g3TRINITY DN37854 c1 g3 i1g.27m.27
TRINITY DN37854 c1 g1TRINITY DN37854 c1 g1 i3g.23m.23
TRINITY DN62062 c0 g1TRINITY DN62062 c0 g1 i1g.36m.36
TRINITY DN16997 c0 g1TRINITY DN16997 c0 g1 i1g.9m.9
TRINITY DN10777 c0 g1TRINITY DN10777 c0 g1 i1g.7m.7
TRINITY DN37854 c1 g1TRINITY DN37854 c1 g1 i6g.31m.31
TRINITY DN37854 c1 g1TRINITY DN37854 c1 g1 i6g.30m.30
TRINITY DN37854 c1 g1TRINITY DN37854 c1 g1 i1g.19m.19
TRINITY DN37854 c1 g1TRINITY DN37854 c1 g1 i1g.18m.18
TRINITY DN37854 c1 g1TRINITY DN37854 c1 g1 i5g.26m.26
TRINITY DN37854 c1 g1TRINITY DN37854 c1 g1 i4g.25m.25
TRINITY DN37854 c1 g1TRINITY DN37854 c1 g1 i8g.34m.34
TRINITY DN37854 c1 g1TRINITY DN37854 c1 g1 i2g.22m.22
TRINITY DN37854 c1 g1TRINITY DN37854 c1 g1 i8g.33m.33
TRINITY DN37854 c1 g1TRINITY DN37854 c1 g1 i2g.21m.21
TRINITY DN3126 c0 g1TRINITY DN3126 c0 g1 i1g.2m.2
